# Plasticity of the Berry Ripening Program in a White Grape Variety

**DOI:** 10.3389/fpls.2016.00970

**Published:** 2016-07-12

**Authors:** Silvia Dal Santo, Marianna Fasoli, Stefano Negri, Erica D'Incà, Nazareno Vicenzi, Flavia Guzzo, Giovanni Battista Tornielli, Mario Pezzotti, Sara Zenoni

**Affiliations:** ^1^Department of Biotechnology, University of VeronaVerona, Italy; ^2^E & J Gallo WineryModesto, CA, USA; ^3^Unione Consorzi Vini Veneti DOCVerona, Italy

**Keywords:** grapevine, berry ripening, plasticity, transcriptomics, metabolomics, phenolic compounds

## Abstract

Grapevine (*Vitis vinifera* L.) is considered one of the most environmentally sensitive crops and is characterized by broad phenotypic plasticity, offering important advantages such as the large range of different wines that can be produced from the same cultivar, and the adaptation of existing cultivars to diverse growing regions. The uniqueness of berry quality traits reflects complex interactions between the grapevine plant and the combination of natural factors and human cultural practices which leads to the expression of wine *typicity*. Despite the scientific and commercial importance of genotype interactions with growing conditions, few studies have characterized the genes and metabolites directly involved in this phenomenon. Here, we used two large-scale analytical approaches to explore the metabolomic and transcriptomic basis of the broad phenotypic plasticity of Garganega, a white berry variety grown at four sites characterized by different pedoclimatic conditions (altitudes, soil texture, and composition). These conditions determine berry ripening dynamics in terms of sugar accumulation and the abundance of phenolic compounds. Multivariate analysis unraveled a highly plastic metabolomic response to different environments, especially the accumulation of hydroxycinnamic and hydroxybenzoic acids and flavonols. Principal component analysis (PCA) revealed that the four sites strongly affected the berry transcriptome allowing the identification of environmentally-modulated genes and the plasticity of commonly-modulated transcripts at different sites. Many genes that control transcription, translation, transport, and carbohydrate metabolism showed different expression depending on the environmental conditions, indicating a key role in the observed transcriptomic plasticity of Garganega berries. Interestingly, genes representing the phenylpropanoid/flavonoid pathway showed plastic responses to the environment mirroring the accumulation of the corresponding metabolites. The comparison of Garganega and Corvina berries showed that the metabolism of phenolic compounds is more plastic in ripening Garganega berries under different pedoclimatic conditions.

## Introduction

The quality traits of grapevine (*Vitis vinifera* L) berries for wine production reflect the outcome of complex interactions between the plant and its environment. In viticulture, the latter is defined as *terroir*, and it represents a combination of natural factors, such as climate, altitude, exposure, the geological characteristics of the soil, and the microbial community, together with human cultural and production practices, which influence the expression of the berry traits and wine quality (White et al., 2009). The combination of a vine and a terroir is unique and is the basis of the term *typicity*, which describes the specific qualitative properties of wines (van Leeuwen et al., 2004). Typicity not only refers to geographically referenced wines but also includes collective taste memory, which has matured for a long time (Vaudour, 2002).

Grapevine is considered one of the most environmentally sensitive crops (Hannah et al., 2013) and it is characterized by broad phenotypic plasticity, i.e., the capacity of a single genotype to express different phenotypes under different environmental conditions (Bradshaw, 1965; Sultan, 2000). Phenotypic plasticity generates variability among berries, clusters, and vines within a vineyard (Dai et al., 2011) and it is often considered a disadvantage in viticulture because it can cause uneven maturity and large inter-seasonal variability (Clingeleffer, 2010). However, genetic variability and plasticity also offer important benefits, such as the large range of different wines that can be produced from the same cultivar, and the adaptation of existing cultivars to specific growing regions (Dai et al., 2011). These phenotypic variations can be attributed to the effect of the environment on the expression of genes influencing plastic traits, mainly through transcriptomic and epigenomic reprograming (Shaw et al., 2014).

Despite the scientific and commercial importance of genotype interactions with growing conditions, few studies have characterized the genes and metabolites directly involved in such phenotypic plasticity. Previously, we reported that ≥5% of the transcriptome of the red berry cultivar Corvina was differentially modulated during berry ripening when comparing plants trained with different systems and rootstocks at sites with diverse pedoclimatic conditions representing a well-known viticultural area in the north of Italy (Dal Santo et al., 2013a). This suggests that the interaction between genotype (grapevine cultivar) and its growing conditions has a profound impact on berry gene expression, possibly affecting ripening and therefore wine quality traits. More recently, Anesi et al. (2015) identified different vine-growers on the basis of the unique metabolic profile of Corvina berries, and Young et al. (2015) described the plasticity of berry carotenoid metabolism in the white variety Sauvignon Blanc under different types of light exposure. These studies revealed few examples of a clear relationship between the plasticity of the berry metabolome and transcriptome, and highlighted the complexity of studying grapevine plasticity in open-field grown plants over multiple growing seasons. Under these conditions, the vines are simultaneously challenged by different external stimuli and are subjected to seasonal waves so that the assignment of a plastic change to a given viticultural practice and/or environmental factor can only be achieved using tailored statistical analysis.

Garganega is a white berry variety mainly cultivated in the Soave production area, which extends into the hills to the east of the Verona province, Italy (Calò et al., 2002). The soil of this production region has both volcanic and alluvial origins, and wines from Garganega berries cultivated in this area are characterized by complexity and longevity with a typical mineral tang. The typicity of Soave wines thus reflects the unique interaction between the Garganega genotype and environmental factors such as the soil origin and composition, and the climatic conditions.

Here, we investigated metabolomic and transcriptomic plasticity during the ripening of Garganega berries representing a single clone cultivated at four sites characterized by different pedoclimatic conditions. To reduce the complexity as far as possible, we selected vineyards trained with similar agricultural practices, but different soil origins and altitudes. We found that the phenotype of the Garganega berries was highly plastic in different environments, indeed more plastic than ripening Corvina berries, particularly concerning the accumulation of phenolic compounds.

## Materials and methods

### Vineyard features and environmental parameters

*Vitis vinifera* cv. Garganega, clone 4, provided by Vivai Coperativi di Rauscedo (VCR) samples were collected from four vineyards during the 2013 growing season at the same time of day (~10.30 a.m.). The parral training system rows were north-south oriented, and SO_4_ was used as the rootstock. For all vineyards, the planting density was 3000–4000 vines/ha, with vines 10–15 years old. The vineyards were located in different areas of the Soave production region featuring diverse growing conditions, such as altitude and soil composition (Table 1). Meteorological data were kindly provided by the Cantina di Soave (Soave, Verona, Italy). Temperature and monthly precipitation (mm) measurements, and the number of rainy days, were obtained from recording stations using the Green Planet Platform (3a S.r.l., Torino, Italy) in the four vineyard sites studied in this project (Colognola ai Colli, Sarmazza di Monteforte d'Alpone, Soave and Ronca'; Supplementary Figure 1). Average daily temperatures were used to define the heat summation per month. Standard soil texture and chemical analysis was conducted by Enocentro Di Vassanelli C. & C. S.r.l. (Verona, Italy).

**Table 1:** **Description of collection sites**.

**Vineyard**	**Location in the soave area**	**Vineyard site**	**Altitude (m a.s.l.)^a^**	**Type of soil(% sand, clay, silt)**	**CaCO_3_(% w/w)^b^**	**Vineyard training system**	**Rows facing direction**	**Vines/hectare**	**Vineyard Age (y)^c^**	**Rootstock^d^**
AP	Colognola ai Colli	45°26′12.90″N, 11°10′30.10″E	84	32.5, 19.9, 47.6	total 22.8 active 16.8	Parral	North-south	3000	10–15	SO_4_
VP	Sarmazza (Monteforte d'Alpone)	45°26′5.27″N, 11°19′2.18″E	32	5.7, 7.5, 86.8	total 3.8 active 2.1	Parral	North-south	4000	10–15	SO_4_
VH1	Ronca'	45°30′9.99″N, 11°17′1.92″E	437	31.4, 10.0, 58.6	total 3.0 active 4.7	Parral	North-south	3000	10–15	SO_4_
VH2	Soave	45°25′28.86″N, 11°12′27.32″E	277	32.4, 4.6, 62.9	total 2.1 active 3.8	Parral	North-south	3500	10–15	SO_4_

a *Meters above sea level*.

b *Dry matter*.

C *Years*.

d *SO, Selection Oppenheim*.

### Sample collection

Garganega berries were collected biweekly at four ripening stages, starting from veraison (the onset of ripening) and finishing at harvest (August 22nd, September 4th, September 17th, and September 30th 2013). For three vineyards (AP, VP, VH2), berries were harvested at the so-called “perfect ripening” stage, corresponding to a total soluble solids (TSS) content of 18.5° Brix and pH 3.3. This was never achieved by the VH1 berries. The °Brix of the berry juice was determined using a digital DBR35 refractometer (Giorgio Bormac S.r.l., Carpi, Italy). A single biological replicate was created by pooling about 30 berries collected from clusters of different vines, along one central vineyard row of an average of 70 vines. As the ripening variability within one single cluster is very large, we payed attention on collecting berries also from different position in the clusters. We repeated the same procedure for the other two biological replicates, but each time we collected berries from different clusters of different vines within the same row. This strategy allowed the collection of three biological replicates that represent almost the entire variability of the vineyard. The same sampling collection procedure was applied at each sampling time point for each of the four vineyards, thus the experiment entailed the collection and analysis of 48 berry samples (four vineyards, four stages, three replicates). We removed the seeds from the berries of each biological replicates and the obtained pericarps were powdered with an automatic mill grinder (IKA®-Werke GmbH & Co. KG, Germany). The obtained frozen powder was used for both transcriptomic and metabolomic analyses.

### RNA extraction and microarray analysis

Total RNA was extracted from ~200 mg frozen berry powder using the Spectrum™ Plant Total RNA kit (Sigma-Aldrich, St. Louis, Missouri, USA) as previously described (Fasoli et al., 2012). A NimbleGen microarray 090818_Vitus_exp_HX12 chip (Roche, NimbleGen Inc., Madison, Wisconsin, USA) was hybridized with 5 μg total RNA per sample according to the manufacturer's instructions. The chip contained probes matching 29,549 predicted grapevine genes (http://ddlab.sci.univr.it/FunctionalGenomics/) representing ~98.6% of the genes predicted in the V1 annotation of the 12x grapevine genome (http://srs.ebi.ac.uk/), as well as 19,091 random probes used as negative controls. Arrays that meet the recommended quality metrics exhibit a background (averaged fluorescence intensity level of empty cells and random probes) of 450–500. Therefore, a fluorescence intensity value of 500 was used as the threshold to define gene expression, and averaged values across the entire dataset lower than 500 were considered to represent minimal/absent expression and were excluded from our analysis.

### Reverse transcription (RT) and real time qPCR

One microgram of extracted RNA was treated with 2 unit (U) of Turbo DNase (TURBO DNA-free kit—Ambion) according to the instructions provided with the commercial kit. DNase-treated RNA was then used for cDNA synthesis using the SuperScript III Reverse Transcriptase kit (Invitrogen) following the producer's indications. In order to assess if the cDNA had been properly produced, an amplification with primers designed on the 3′ UTR of an Ubiquitin coding gene (VIT_16s0098g01190; UbiFor 5′-TCTGAGGCTTCGTGGTGGTA-3′ and UbiRev 5′-AGGCGTGCATAACATTTGCG-3′) was performed.

Real Time qPCR was performed using GoTaq® Green Master Mix kit (Promega) to amplify a specific region of target genes (C4H, trans-cinnamate 4-monooxygenase, VvC4H-F: 5′-AAAGGGTGGGCAGTTCAGTT-3′ and VvC4H-R: 5′-GGGGGGTGAAAGGAAGATAT-3′; MYB14, transcription factor MYB14, VvMYB14-F: 5′-TCTGAGGCCGGATATCAAAC-3′ and VvMYB14-R: 5′-GGGACGCATCAAGAGAGTGT-3′; ANR, anthocyanidin reductase, VvANR-F: 5′-CAATACCAGTGTTCCTGAGC-3′ and VvANR-R: 5′-AAACTGAACCCCTCTTTCAC-3′; LAR1, leucoanthocyanidin reductase 1, VvLAR1-F: 5′-CACATGCATGCGATTAGTCC-3′ and VvLAR1-R: 5′-ACGAATTTCACCCATGTTAC-3′). Reaction mix was composed of 1x GoTaq® Green Master Mix, 200 nM of each primer and 20 ng of cDNA in a final volume of 25 μl. The reaction was carried out on a StepOnePlus™ Real Time PCR System (Applied Biosystems) using the following cycling conditions: 95°C hold for 2 min followed by 40 cycles at 95°C for 15 s, 55°C for 30 s, 60° for 30 s, and 95°C for 15 s. Non-specific PCR products were identified by the dissociation curves. Amplification efficiency was calculated from raw data using LingRegPCR software (Ramakers et al., 2003). The mean normalized expression (MNE)-value was calculated for each sample referred to the ubiquitin expression according to the Simon equation (Simon, 2003). Standard error (*SE*)-values were calculated according to Pfaffl et al. (2002).

### Statistical analysis of the transcriptome dataset

Principal component analysis (PCA) was applied to the entire transcriptome dataset using SIMCA P+ v13 (Umetrics, San José, California, USA). Loadings of the first and second principal components were ordered, and genes within the first and last percentiles were extracted to investigate their expression profiles over time in the different vineyards. Multiclass significance analysis of microarrays (SAM) was carried out using TMeV v4.8 (http://www.tm4.org/) with a false discovery rate (FDR) of 0.01%, to extract genes that were significantly modulated during ripening in all four vineyards. The differentially expressed genes were then filtered by applying a fold-change thresholds of ≥2 or ≤ −2. To identify shared and specific transcriptomic ripening programs in the four vineyards, the selected differentially expressed genes (FDR 0.01%, |FC| ≥2) were represented in a Venn diagram (Venny v2.0, http://bioinfogp.cnb.csic.es/tools/venny/). The differentially expressed genes were screened by calculating the coefficients of variation (CV) at the four developmental stages in each vineyard, and then the standard deviation (*SD*) among the four calculated CVs. This allowed to rank the shared genes by a quantitative measure of the intra-vineyard and inter-vineyard variability of expression during ripening. Transcripts scoring the highest standard deviations (the top 50 genes) were defined as the most plastic genes under our experimental conditions.

### Functional category assignments and GO enrichment analysis

All grapevine transcripts were annotated against the V1 version of the 12x draft annotation of the grapevine genome. Gene Ontology annotations were assigned using the BiNGO v2.3 plug-in tool in Cytoscape v2.6 (http://www.cytoscape.org/) with PlantGOslim categories. Overrepresented PlantGOslim categories were identified using a hypergeometric test with a significance threshold of 0.05, after Benjamini and Hochberg correction with a FDR of 0.01 (Klipper-Aurbach et al., 1995).

### Visualization of grapevine transcriptome data

Information from the Nimblegen microarray platform was integrated using MapMan software (Thimm et al., 2004) as described for the Array Ready Oligo Set *Vitis vinifera* (grape), the AROS V1.0 Oligo Set (Operon, Qiagen, Hilden, Germany), and the Gene-Chip® *Vitis vinifera* Genome Array (Affymetrix Inc., Santa Clara, California, USA; Rotter et al., 2009). Mapping information and the annotation of the carotenoid biosynthesis and catabolic pathways were modified based on Young et al. (2012).

### Extraction, analysis, and identification of non-volatile metabolites

For each sample, 300 mg of frozen berry powder was extracted in three volumes of cold methanol acidified with 0.1% formic acid. After mixing, the samples were sonicated for 15 min at 4°C and then centrifuged at 16,000 × *g* for 10 min at 4°C. The supernatants were analyzed by reversed-phase high-performance liquid chromatography (RP-HPLC) coupled to electrospray ionization mass spectrometry (RP-HPLC-ESI-MS) or a diode array detector (RP-HPLC-DAD) after dilution (1:2 or 2:3, respectively) in LC-MS-grade water and passage through a 0.2-μm filter.

RP-HPLC analysis was carried out using a Beckman Coulter (Brea, California, USA) Gold 127 HPLC System equipped with a C18 guard column (7.5 × 2.1 mm, 5 μm particle size) and an Alltech (Nicholasville, Kentucky, USA) RP C18 column (150 × 2.1 mm, 3 μm particle size). Two solvents were used: 0.5% formic acid and 5% acetonitrile in water (solvent A) and acetonitrile (solvent B). The gradient was set as follows: 0–10% B in 2 min, 10–20% B in 10 min, 20–25% B in 2 min, 25–70% B in 7 min, isocratic for 5 min, 70–90% B in 1 min, isocratic for 4 min, 90–0% B in 1 min, and 20 min equilibration. For each sample, 20 μL was injected at a flow rate of 0.2 mL min^−1^. The HPLC instrument was coupled on-line with an Esquire 6000 ion trap mass spectrometer equipped with an ESI source (Bruker Daltonik GmbH, Bremen, Germany). MS data were collected using the Esquire Control v5.2 software, and processed using the Esquire Data Analysis v3.2 software (both provided by Bruker Daltonik GmbH). The instrument was set to induce positive and negative ionization in alternating mode. Mass spectra were recorded in the range 50–3000 m/z with a target mass of 400 m/z. MS/MS and MS^3^ mass spectra were recorded in positive and negative ionization modes in the range 50–3000 m/z with a fragmentation amplitude of 1 V. Nitrogen was used as the nebulizing gas (50 psi, 350°C) and drying gas (10 L min^−1^). Helium was used as the collision gas. The vacuum pressure was 1.4 × 10^−8^ bar. Additional parameters: capillary source 4500 V; end plate offset –500 V; skimmer: 40 V; cap exit 121 V.

RP-HPLC-DAD analysis was carried out using a Beckman Coulter Gold 126 Solvent Module and Gold 168 Diode Array Detector. HPLC was carried out as above, with a larger injection volume of 100 μL. The wavelength range was 190–600 nm. Data were collected and analyzed using 32 Karat v7.0 (Beckman Coulter). Specific wavelengths were considered to represent the following classes of compounds: 280 nm (flavan-3-ols and their oligomers), 320 nm (hydroxycinnamic acid derivatives), and 350 nm (flavonols).

Metabolites were identified by comparing their retention times, m/z-values and MS^n^ fragmentation patterns with those of commercial standards in our in-house library. UV/vis spectra recorded by RP-HPLC-DAD were also used to support the LC-MS identification. Fragmentation patterns collected in online databases such as MassBank (http://www.massbank.jp) or reported in the literature were also considered, especially when no authentic standard compounds were available. Neutral losses of 132, 146, and 162 Da were considered diagnostic of the loss of pentose, deoxyhexose, and hexose sugar, respectively.

### LC-MS data processing and statistical analysis

LC-MS chromatograms were converted to netCDf files for peak alignment and area extraction using MZmine (http://mzmine.sourceforge.net/). The resulting matrix was analyzed using SIMCA v.13.0 (Umetrix AB, Umea, Sweden). Pareto scaling was applied to all analytical methods. Unsupervised PCA was used to identify the major clusters defined by the samples prior to supervised partial least squares discriminant analysis (PLS-DA) and orthogonal partial least squares discriminant analysis (OPLS-DA and O2PLS-DA) setting the classes according to the ripening stage for each vineyard location. PLS-DA models were validated using a permutation test (200 permutations) and the corresponding OPLS-DA/O2PLS-DA models were cross-validated by analysis of variance (ANOVA) with a threshold of *p* < 0.01.

### Accession numbers

Grape berry microarray expression data are available in the Gene Expression Omnibus under the series entry GSE75565 (http://www.ncbi.nlm.nih.gov/geo/query/acc.cgi?acc=GSE75565). The datasets supporting the Garganega metabolome analysis are included in this article and its supplementary files. The Corvina metabolomics data are available in the Metabolights database under the series entry MTBLS39.

## Results

### Pedoclimatic conditions influence the garganega berry ripening process

**Figure 1:**
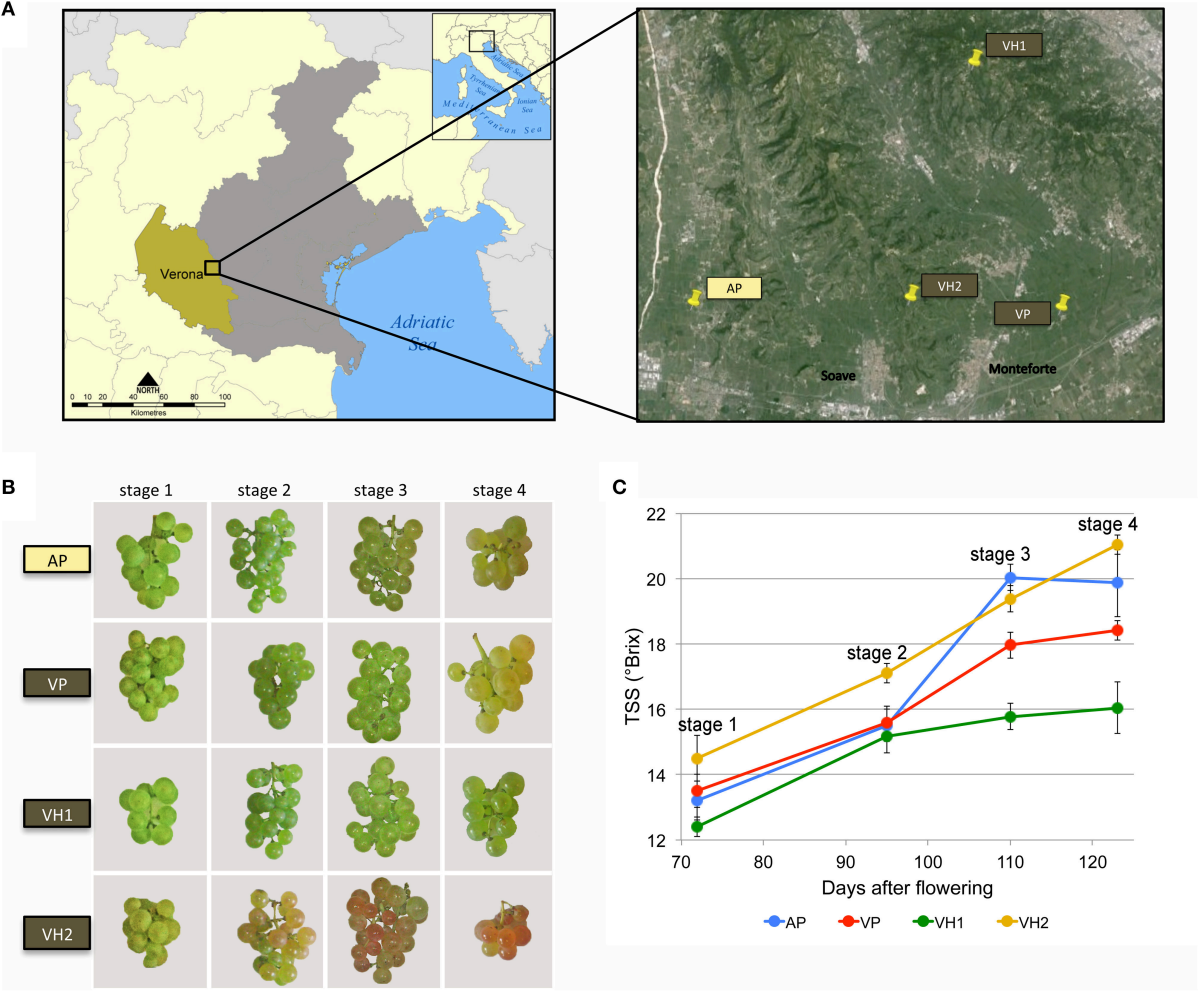
**Overview of the Garganega berry samples used for transcriptome and metabolome analysis. (A)** Sampling locations near Verona, Italy. Four different vineyards were chosen from the Soave wine production macro-area. AP (Alluvial Plain, yellow box); VP (Volcanic Plain, brown box); VH1 (Volcanic Hill 1, brown box); VH2 (Volcanic Hill 2, brown box). **(B)** Images of biweekly samples showing Garganega grape cluster maturation in four vineyards. **(C)** Grape berry ripening as shown by sugar accumulation from veraison through to harvest (four stages) in the four vineyards. TSS = total soluble solids.

*Vitis vinifera* cv. Garganega clone four berries were harvested from four different vineyards surrounding the Soave area, one of the most important wine production macro-areas in the province of Verona, Italy (Figure 1A). The vineyards were selected to maximize differences in environmental conditions (altitude and soil type) while minimizing differences in agricultural practices (training system, orientation of the rows, planting layout, vineyard age, and rootstock type; Table 1). The selected vineyards were characterized by a hill (VH1 and VH2) or plain (AP and VP) altitudinal position, and by an alluvial (AP) or volcanic (VP, VH1, and VH2) soil type (Table 1 and Figures 1A,B). The alluvial area (AP) featured almost twice the proportion of clay particles in the soil compared to the volcanic sites, among which the plain site (VP) was characterized by a silty soil with the lowest percentage of sand. VH1 and VH2 had similar soil textures, despite being located at different altitudes. The calcium carbonate component (total and active) was much lower in the VP, VH1, and VH2 vineyard sites than in the more calcareous alluvial AP site (Table 1). This is typical for “ando soils,” which developed on volcanic ash. Meteorological parameters were recorded, and the daily temperature data showed that more heat accumulated (heat summation per month) in the hillside areas in July, August, and September, but overall the heat summation was similar across all four vineyards (Supplementary Figure 1). Rainfall data showed greater variability among the areas albeit with no significant differences in terms of mm of rain per year, however VH2 was a more rainy area during the growing period (April 1st to October 31st in the Northern Hemisphere). Berry samples were harvested from all vineyards on the same day and three biological replicates were taken at each of the four developmental stages (Figure 1B). The TSS content was verified by measuring °Brix values (Figure 1C). Although the sampling date was aligned to the first developmental stage, the dynamics of TSS accumulation were unique to each vineyard. Interestingly, the two hillside vineyards showed the most divergent behavior: VH2 showed a steady increase in the TSS content whereas VH1 scored lowest for the accumulation of TSS.

### Unraveling the metabolome of garganega ripening berry

**Figure 2:**
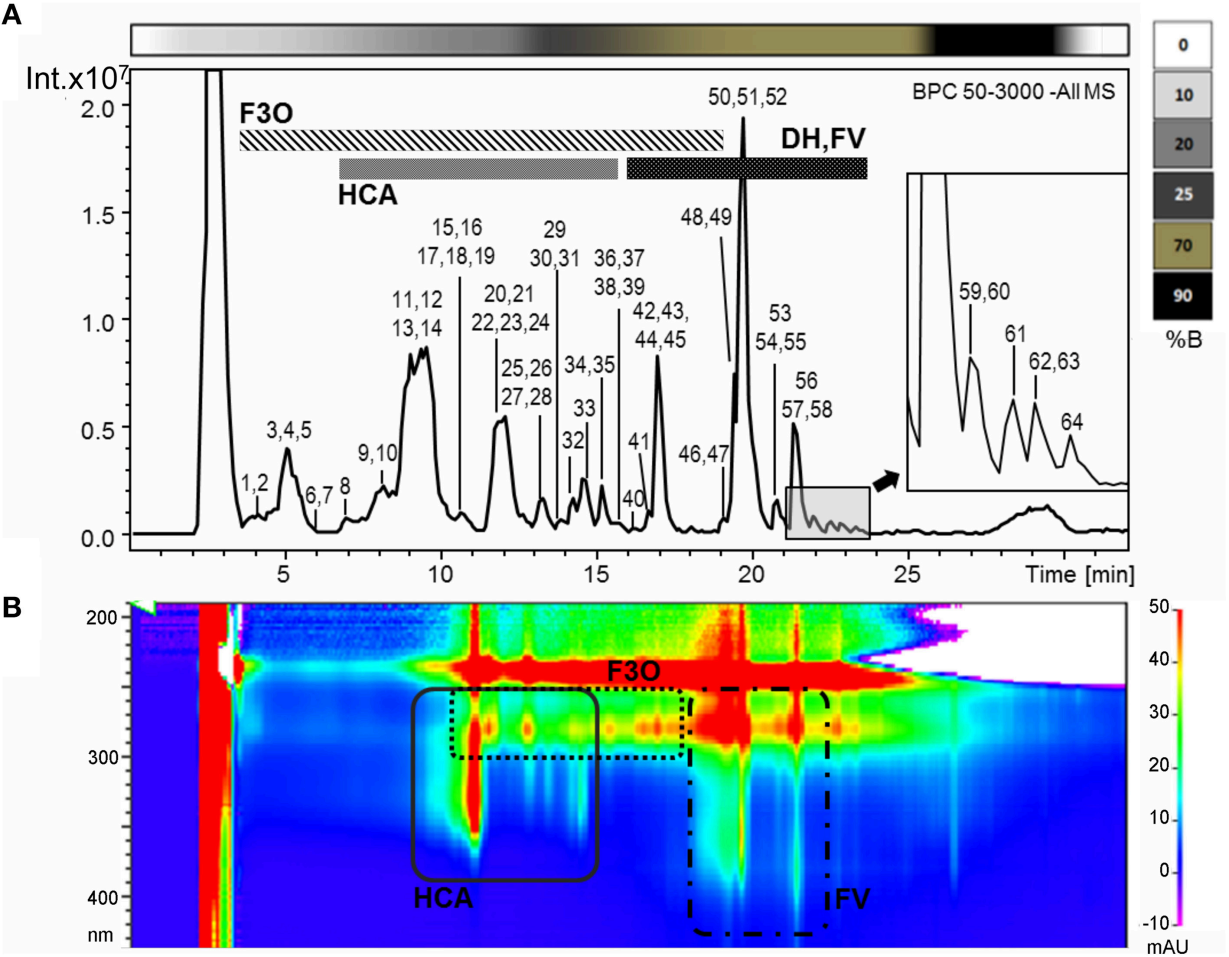
**(A)** Base peak chromatogram of a Garganega methanolic extract recorded in negative ionization mode during RP-HPLC-ESI-MS analysis. The peaks numbers correspond to the metabolites identified in the Supplementary File 1 (column A) **(B)** Bidimensional RP-HPLC-DAD chromatogram recorded within the wavelength range 190–600 nm. F3O (flavan-3-ols and oligomers), HCA (hydroxycinnamic acid derivatives), DH (dihydroflavonol glycosides), FV (flavonol glycosides). The upper bar refers to the percentage of solvent B within the mobile phase.

Untargeted RP-HPLC-ESI-MS analysis was used to characterize the Garganega berry metabolome at each of the four vineyards (AP, VP, VH1, VH2) at four ripening stages, focusing on moderately polar metabolites such as phenolic compounds. Data processing revealed 267 signals in negative ionization mode (Figure 2). The comparison of the fragmentation patterns with an in-house library of authentic standards and literature data led to the putative identification of 64 metabolites mainly representing the flavan-3-ols and their oligomers, phenolic acids, flavonol, and dihydroflavonol glycosides (Figure 2, Supplementary File 1). The major flavan-3-ol monomers were catechin (the most abundant) followed by epicatechin and two epigallocatechin isomers, whereas different types of oligomers were detected, including dimeric and trimeric procyanidins and prodelphinidins. Two galloylated derivatives were also identified as epicatechin gallate and procyanidin dimer gallate, and one compound was tentatively identified as a glycosilated procyanidin dimer (Maldini et al., 2009).

Another large group of metabolites corresponded to the hydroxycinnamic acids. Within this group, caffeoyltartaric acid was the most abundant, followed by coumaric and ferulic acid glycosides and tartaric acid esters (coutaric and fertaric acid). Minor signals were also assigned to the hexose esters of three benzoic acids (syringic, vanillic, and gallic acid).

Among the flavonols, the glycosides of quercetin, kaempferol, isorhamnetin, and myricetin were detected, as well as those of the dihydroflavonols, dihydroquercertin, and dihydrokaempferol. Other minor compounds were putatively identified, including two isomers of resveratrol glucoside, the phenylethanoid hydroxytyrosol glucoside, and an N-conjugated glycoside of tryptophan.

### The four vineyards show different trends in the accumulation of specific metabolites during ripening

**Figure 3:**
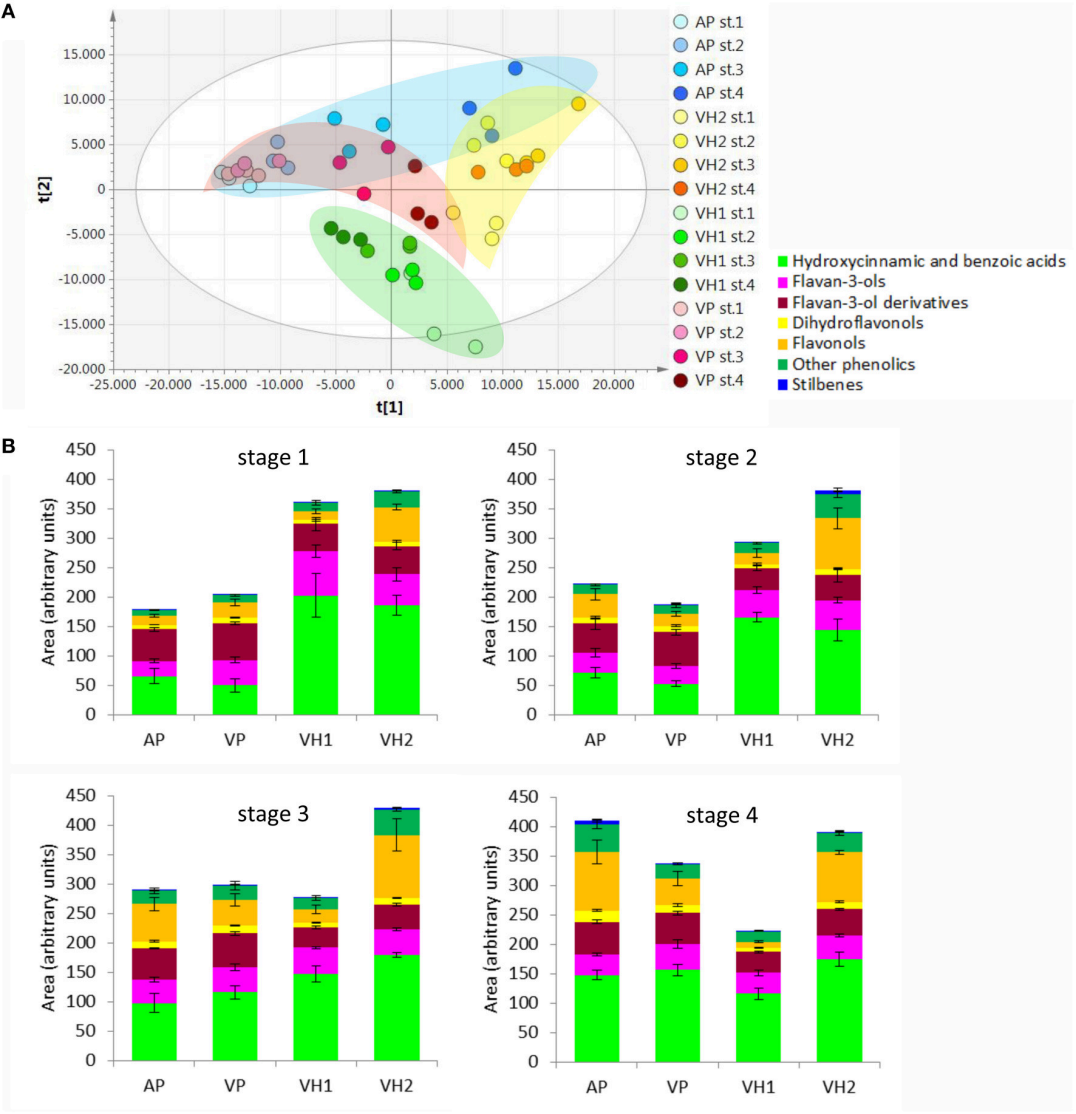
**(A)** PCA score scatter plot representing the dataset of metabolites detected by RP-HPLC-ESI-MS analysis. **(B)** Relative comparison of the levels of phenolic compounds detected in the Garganega samples by RP-HPLC-ESI-MS at the four ripening stages.

The accumulation of metabolites among the four vineyards during ripening was initially compared by inspecting the entire data matrix by unsupervised multivariate PCA. Two partially overlapping clusters were observed for the VP and AP samples, whereas the VH1 and VH2 samples formed two independent clusters (Figure 3A). This clustering suggested that the two plain vineyards (AP, VP) shared similar metabolomes during the earlier stages of ripening, with differences emerging only during the last stage. In contrast, the two hillside vineyards (VH1, VH2) were characterized by distinct metabolomes from the earlier stages. These trends were confirmed by the comparison of phenolic compounds in the samples, revealing that the plain and hillside vineyards were characterized by different metabolomes throughout the ripening process (Figure 3B). These findings suggested that the Garganega berry metabolome is modulated during ripening according to the location of the vineyards.

**Figure 4:**
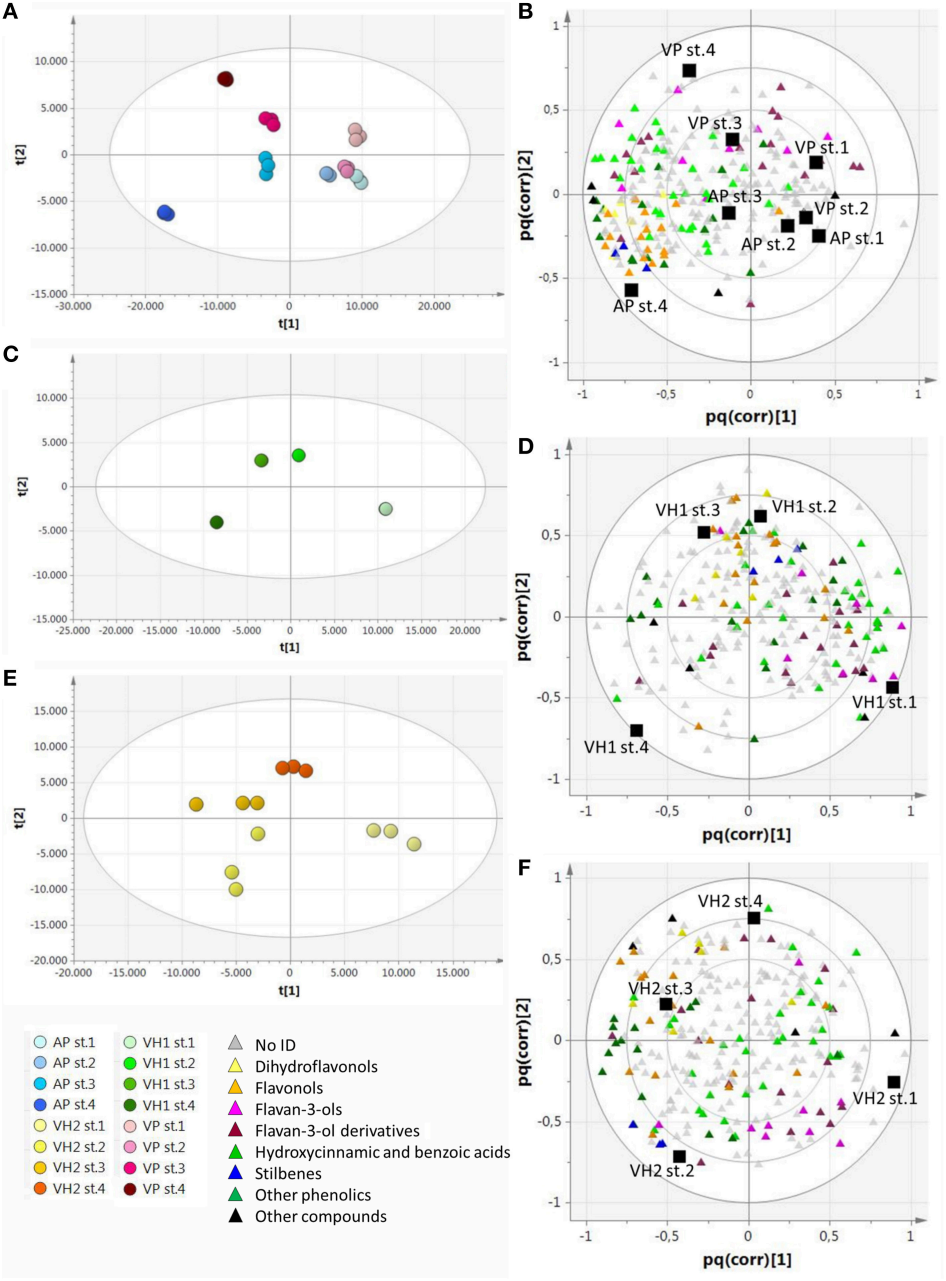
**O2PLS-DA score plots and correlation loading plots for the metabolites detected by RP-HPLC-ESI-MS analysis. (A,B):** AP and VP samples; **(C,D)**: VH1 samples; **(E,F)**: VH2 samples. Sample clusters and groups of metabolites are depicted in different colors as shown in the legend.

In order to find metabolites that characterized the various groups of samples, we applied a supervised O2PLS-DA approach (Figure 4). The plain vineyards were analyzed together due to their similar behavior in the PCA, and this revealed an enrichment of metabolites (especially hydroxycinnamates) during ripening, with the dihydroflavonols and flavonols becoming particularly characteristic of the AP vineyard at stage 4 (Figures 4A,B). The hillside vineyard VH1 showed the opposite trend, with many metabolites (particularly flavan-3-ols and their oligomers and phenolic acids) decreasing at the end of ripening. Dihydroflavonols and flavonols peaked during the third ripening stage and decreased toward the end of ripening, which is generally indicative of poor ripening and suggests that an unknown event inhibited the ripening process (Figures 4C,D). Flavan-3-ols and their oligomers characterized the first two ripening stages in the hillside vineyard VH2, but the dihydroflavonols and flavonols peaked during the third stage as described above (Figures 4E,F).

### The garganega transcriptome dataset also reveals differences between vineyards

**Figure 5:**
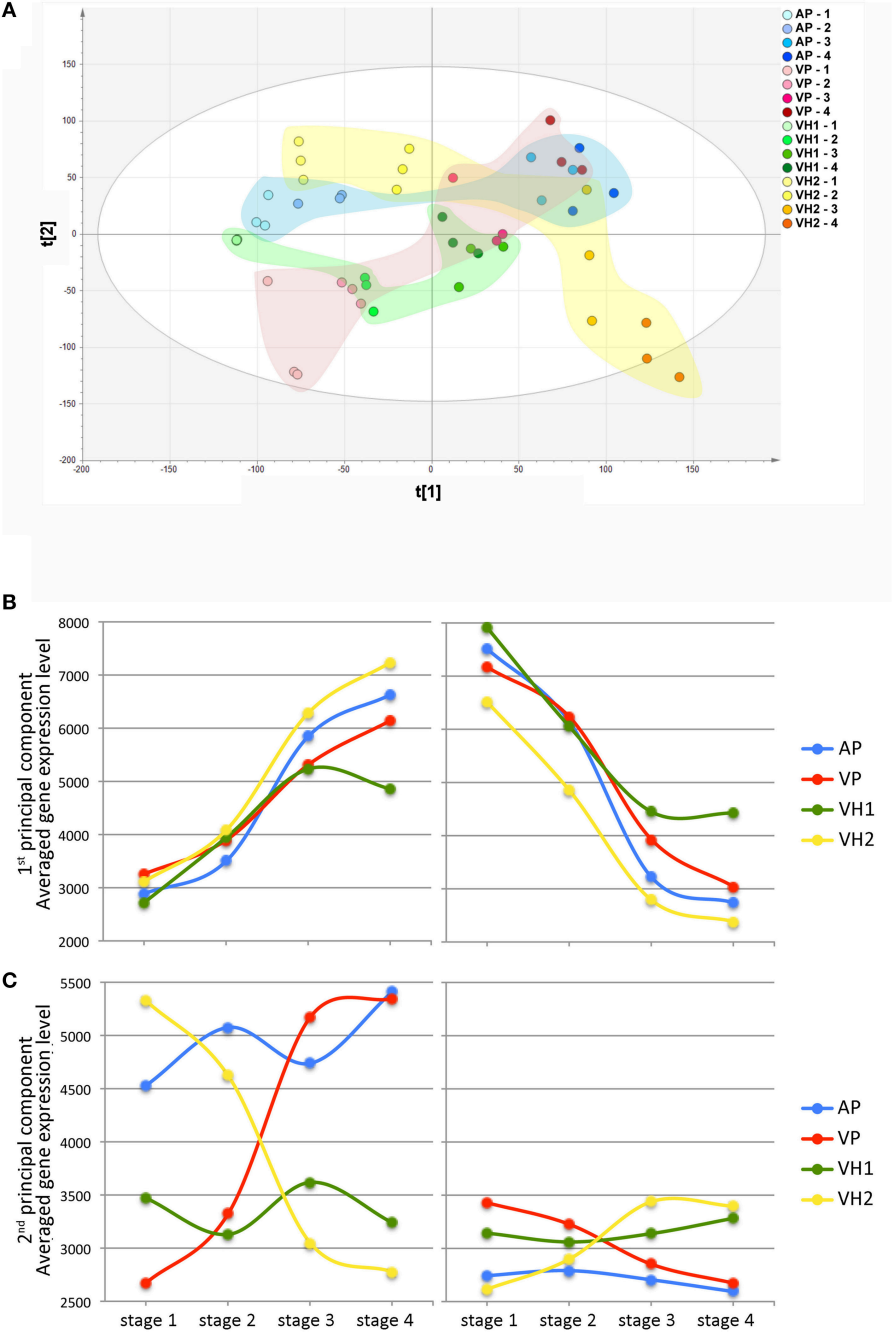
**Global gene expression trends in Garganega berries cultivated at four locations during ripening. (A)** Variables and scores scatterplot of the PCA model (R2X = 0.748, Q2cum = 0.582) applied to the entire dataset. The expression profiles of genes positively (left) and negatively (right) correlated to the first **(B)** and second **(C)** principal components were selected within the first (positive) and the last (negative) percentile of each of the component loadings.

Following microarray hybridization, the pericarp transcriptome dataset of the ripening Garganega berries was inspected by PCA, confirming the consistency of the biological replicates (Figure 5A). PC1 explained 27.6% of the total variability and was attributed to differences in the ripening stage among the samples. Despite the small difference in PC1 at the first sampling point, the dynamics of ripening differed among the vineyards at the level of the transcriptome (Figure 5A). In particular, VH1 was characterized by a clear interruption of the ripening process, whereas VH2 reached a more advanced ripening stage. These differences corresponded to the increase in °Brix values in the VH1 and VH2 vineyards (Figure 1C) and strongly suggested that VH1 never reached the “full ripening” stage.

Differences among the vineyards were highlighted by visualizing the average trend of the first and last percentiles of the PC1 loadings (Figure 5B). As expected, the averaged trends of genes representing the first percentile increased during ripening, whereas those of genes representing the last percentile decreased. In both cases, it was possible to rank the final ripening level reached in each vineyard on the basis of the averaged gene expression level at the final time point (VH2 > AP > VP > VH1). Interestingly, there was a larger difference in the expression level of the last percentile of the PC1 loadings compared to the first percentile of the PC1 loadings at the first time point (Figure 5B). This could explain the slight difference among samples at the first time point revealed by PC1 (Figure 5A), suggesting that the onset of ripening was predominantly defined by the downregulation rather than the upregulation of genes. We found that 55 genes among the positively-correlating PC1 loadings (26.96%) are already described as putative master regulators of ripening in five red berry varieties (Palumbo et al., 2014). In contrast, many photosynthesis-related genes were found among the negatively-correlating PC1 loadings, confirming that the suppression of photosynthesis is one of the main events driving the berry toward maturation (Fasoli et al., 2012; Supplementary File 2).

PC2 explained 16.5% of the total variability (Figure 5A) and mainly describes differences between vineyards. Such differences were already evident at the first time point, and showed that VH2 and VP were the two most distant conditions. Interestingly, these two vineyards followed opposite trends along PC2 during ripening, resulting in well-separated final stages. In contrast, only minor variations along PC2 were observed for the AP and VH1 vineyards during ripening (Figure 5A). These findings are well-supported by the averaged trends of the first and last percentiles of the PC2 loadings (Figure 5C). Major changes and opposing trends in gene expression were observed for VP and VH2, whereas AP and VH1 showed different averaged levels of gene expression but little change during ripening. Interestingly, the PC2 first percentile loadings were expressed at a higher level overall than the last percentile loadings, suggesting the first percentile made the major contribution to the variability described by PC2.

The functional categories of the PC2 first percentile genes indicated that Carbohydrate metabolism, DNA, and RNA metabolic processes (transport, surveillance, and degradation) and Transport were key processes (Supplementary File 2). Only a few genes related to Secondary metabolism were found, including three encoding cinnamyl alcohol dehydrogenases (CADs). In contrast, the PC2 negative loadings were rich in Transcription factors, Pentatricopeptide repeat (PPR) proteins, and proteins related to Cellular homeostasis (Supplementary File 2).

### Differences in gene expression reflect different characteristics of the four vineyards

**Figure 6:**
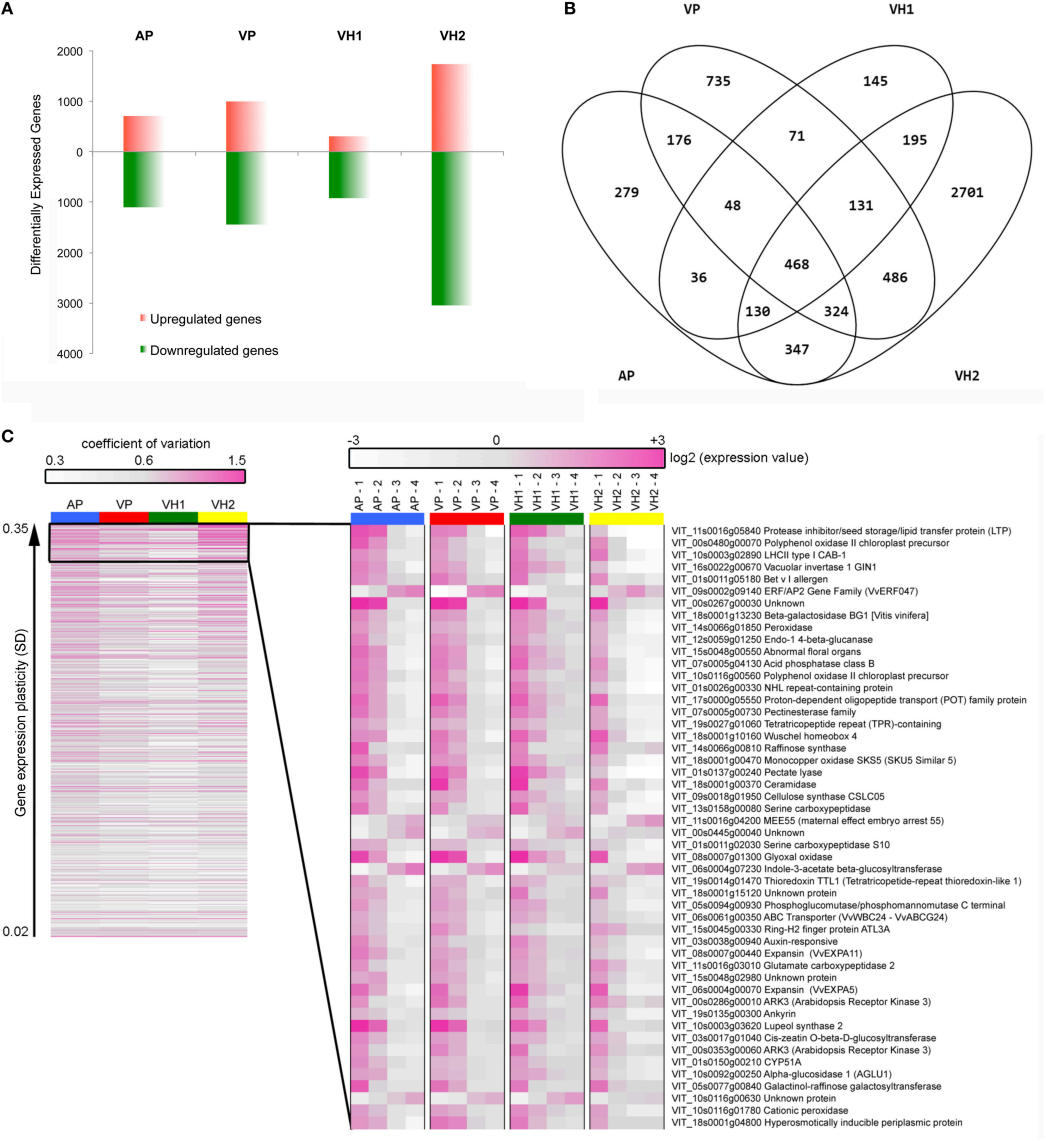
**Grapevine transcripts showing modulation and plasticity during Garganega berry maturation within the four vineyards. (A)** Number of genes significantly upregulated or downregulated during ripening. **(B)** Shared and specific gene expression modulation. **(C)** Heat maps of the 468 core genes represented by the coefficient of variation among the four ripening stages (left) and of the top 50 most plastic transcriptional profiles using the average expression value of three biological replicates (right).

The plasticity of the ripening Garganega berry transcriptome in the four different vineyards was investigated by multiclass statistical analysis of microarrays (SAM) within each group of vineyard samples. A total of 12,931 transcripts were significantly modulated (Supplementary File 3) and, of these, 6272 scored a fold change (|FC|) ≥2 in at least one condition. This revealed that vineyard VH2 featured the highest number of modulated genes (4782) and vineyard VH1 the lowest number (1224). In vineyards AP and VP, the number of differentially modulated genes with a |FC| ≥2 was 1808 and 1441, respectively (Supplementary File 3 and Figure 6A). Interestingly, all four vineyards were characterized by a higher number of downregulated genes than upregulated genes (Figure 6A), confirming that berry ripening predominantly involves gene suppression rather than activation (Palumbo et al., 2014).

The significantly modulated genes were either specific or common among the four different vineyards (Supplementary File 3 and Figure 6B). The VH2 vineyard featured the greatest number of specifically modulated genes, i.e., 56.48% of the VH2 modulated genes and 43.04% of all the modulated genes (Figure 6B). These genes were particularly enriched in functional categories related to stress, such as Death, Cell death, and Response to stress, as well as the categories Protein modification and Cell communication (Supplementary Figure 2). The 2701 genes specifically modulated in vineyard VH2 also included those encoding a large number of R proteins, other disease resistance proteins, and heat shock proteins, as well as 25 glutathione-S-transferases (GSTs), 16 CADs, and many terpene synthases (TPSs) involved in the production of distinct volatile compounds. Up to 90 genes encoding PRR-containing proteins were also found in this class (Supplementary File 3). PRR-containing proteins are thought to be involved in RNA metabolism (Barkan and Small, 2014) and show a high level of plasticity in Corvina berries (Dal Santo et al., 2013a). Many transporters, including 13 ABC transporters (Çakır and Kilickaya, 2013) and signal transduction-related transcripts, were found among the VH2-specific modulated genes. Interestingly, genes encoding nine histone proteins, a histone acetyltransferase (HAC1), and three histone-lysine N-methyltransferases were specifically modulated in the VH2 vineyard during berry ripening, suggesting that histone modifications could play an important role in the plasticity of the Garganega berry transcriptome. Some of the VH2-specific transcripts also represented enzymes involved in carotenoid metabolism, i.e., *VvAAO3, VvLECY1, VvLBCY1, VvZEP2*, and *VvBCH1* (Young et al., 2012).

The transcriptional plasticity of carotenoid-related genes in ripening Garganega berries was investigated in more detail by screening the 12,931 modulated transcripts (Supplementary File 3) and visualizing the genes involved in carotenoid synthesis and catabolism (Young et al., 2012) using the MapMan heat map representation (Supplementary Figure 3). Many genes in the carotenoid pathway were significantly modulated in one or more of the vineyards: VH2 featured 22 such genes, the highest number, VP and AP (both located on plains) featured eight and seven, respectively, and VH1 featured five. Genes representing all branches of the pathway were expressed in VH2, including the common pathway to lycopene, the lutein branch, the β-carotene branch, abscisic acid (ABA) biosynthesis and degradation, and the cleavage of mature carotenoids to form apocarotenoids and strigolactone (Supplementary Figure 3). Only one carotenoid-related gene was significantly expressed in all four vineyards during berry ripening, i.e., *VvNCED3* representing the most important enzyme in the ABA biosynthesis pathway (Sun et al., 2010).

VH1 featured the lowest number of specifically modulated genes, i.e., 11.85% of the VH1 modulated genes and 2.31% of all the modulated genes (Figure 6B). Interestingly, GO analysis revealed the enrichment of the categories Secondary metabolic processes and Biosynthetic processes (Supplementary Figure 2). In particular, 15 stilbene synthases (which synthesize resveratrol) and other genes representing the phenylpropanoid/flavonoid pathway were found among the VH1-specific genes (Supplementary File 3).

VP featured 735 specifically-modulated genes, i.e., 30.13% of the VP modulated genes and 11.72% of all the modulated genes (Figure 6B). GO analysis revealed the enrichment of the categories Death, Cell death, Lipid metabolic processes, and Cell communication (Supplementary Figure 2). Indeed, many genes involved in fatty acid biosynthesis, as well as those encoding R proteins, disease resistance proteins, and transporters, were found among the VP-specific modulated genes (Supplementary File 3).

Finally, AP featured 279 specifically-modulated genes, i.e., 15.43% of the AP modulated genes and 4.44% of all the modulated genes (Figure 3B). No significant GO category enrichment was revealed by BINGO analysis.

### The commonly modulated portion of the transcriptome contains plastic transcripts

A core of 468 genes (7.46% of all modulated genes as shown in Figure 6B) was found to represent the shared portion of the transcriptome, which was modulated in ripening Garganega berries regardless of the vineyard (Supplementary File 3). Interestingly, 65 core genes were also defined as switch genes, which are proposed to drive berries of five Italian red varieties from vegetative growth into the ripening phase (Palumbo et al., 2014). BINGO analysis revealed significant enrichment of the categories Photosynthesis, Generation of precursor metabolites and energy, and Carbohydrate metabolic processes (Supplementary Figure 2). Indeed, five genes representing the photosystem light harvesting complexes were found among the shared transcripts as well as many genes involved in specific carbohydrate metabolic processes, such as galactose, starch, and sucrose metabolism. The acidic vacuolar invertase *VvGIN1* and the sugar symporter *VvSUC2* were found among the common core genes (Supplementary File 3), and their expression increased simultaneously with post-veraison sugar accumulation (Davies and Robinson, 1996; Davies et al., 1999; Afoufa-Bastien et al., 2010).

After veraison, auxin levels decline sharply (Davies et al., 1997). Accordingly, many of the commonly modulated genes we identified are involved in auxin biosynthesis, transport, and signaling (Supplementary File 3), and others are related to cytokinins, ethylene, brassinosteroids, and salicylic acid. Furthermore, 39 of the genes were annotated as transcription factors, three of which have already been described as putative master regulators of berry ripening: LBD18, Myb TKI1, and VvNAC11 (Palumbo et al., 2014). Finally, 52 transporters were significantly modulated in all four vineyards, indicating that intracellular transport plays a crucial role during the ripening of Garganega berries (Supplementary File 3).

We next analyzed the expression profiles of all 468 shared core genes to evaluate their transcriptional plasticity during berry development in the four vineyards. The heat map in Figure 6C (left panel) clearly shows that vineyard VH2 displayed the highest variability among the four developmental stages, followed by vineyards AP and VP, whereas the dynamic range of gene expression in vineyard VH1 was more attenuated. We next focused on the top 50 genes scored by SD (Figure 6C, right panel). Most of these genes were downregulated, whereas only five were upregulated in ripening berries. The latter encoded ERF/AP2 transcription factor 47 (VvERF047), the maternal-effect embryo arrest protein 55, an indole-3-acetate β-glucosyltransferase, and two unknown proteins. The patterns of downregulation showed that the ripening process was delayed in vineyard VH1, was similar in the two vineyards located on plains (AP and VP) and was accelerated in VH2, confirming the °Brix trends (Figure 1C). For example, the expression of the photosystem light harvesting complex LHCII and the vacuolar invertase VvGIN1 declined sharply after veraison in VH2 but declined gently from veraison to harvest in the other three vineyards. Many genes in the Cell wall metabolism category were found among the most plastic common genes expressed as described above, including a β-galactosidase, an endo-1,4-β-glucanase, a pectinesterase, a pectate lyase, the CSLC05 type cellulose synthase, and two expansins. Notably, the two expansin genes (*VvEXPA5* and *VvEXPA11*) have already been reported as markers of the veraison phenological phase (Dal Santo et al., 2013b) confirming that berry ripening was more advanced in VH2 than the other vineyards.

### A comparison between garganega and corvina transcriptome plasticity

In a previous study we evaluated the berry transcriptome plasticity of the red berry variety Corvina clone 48, through three consecutive growth years cultivated in 11 different vineyards in the Verona area (Dal Santo et al., 2013a). In order to compare the plasticity within red and white berry transcriptome during ripening, we chose four out of the 11 vineyards, basing our decision on the location (i.e., Soave and Valpolicella wine growing regions) and on the berry ripening stage (Supplementary File 4). This Corvina 36-sample reduced dataset (four vineyards, three developmental stages, three biological replicates) was then processed using the same statistical procedure described above for the Garganega berry transcriptome. We found that 2894 Corvina genes were significantly modulated (Supplementary Figure 4 and Supplementary File 5), representing 46.14% of all modulated genes in Garganega berries. This suggested that the white variety transcriptome could be modified to a greater extent by the ripening process and/or by the growing conditions compared to the red variety transcriptome.

We next compared the developmentally-regulated portion of each transcriptome focusing on the environmentally-sensitive genes, i.e., those genes expressed in one of the four selected vineyards. In the Corvina cultivar we identified 2021 genes representing 69.83% of all modulated Corvina genes whereas in the Garganega cultivar we identified 3860 genes representing 61.51% of all modulated Garganega genes (Supplementary Figure 4). Therefore, despite the significant difference in the total number of modulated genes during ripening, the vineyard-specific portion of the transcriptome was approximately the same size in the red and white berry varieties. Specifically, the Garganega and Corvina varieties shared 409 genes (Supplementary File 6), which were particularly enriched in the GO categories Carbohydrate metabolic processes, Secondary metabolic processes, Lipid metabolic processes, and Photosynthesis (Supplementary Figure 5). These categories are therefore likely to be the most strongly influenced by the environment and may encompass the largest number of environmentally-sensitive genes.

### The phenylpropanoid pathway is more plastic in the white berry variety

**Figure 7:**
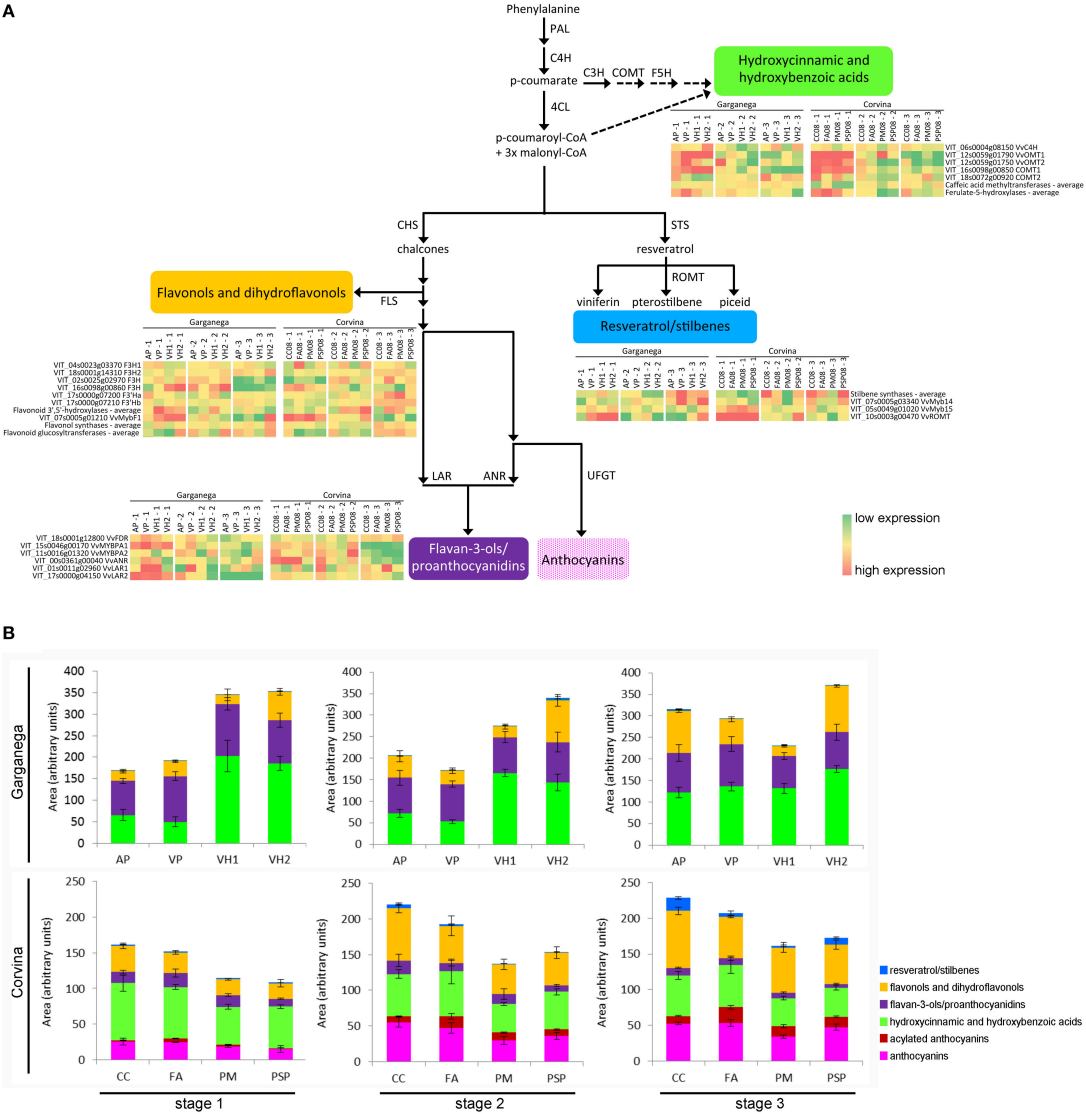
**Comparison of Corvina and Garganega transcriptomic and metabolomic changes in the general phenylpropanoid pathway during berry ripening**. The third and fourth Garganega ripening stages were averaged for gene expression and the levels of phenolic compounds to facilitate alignment with the three available Corvina ripening stages and their corresponding °Brix values (Supplementary File 4) **(A)** Simplified representation of the general phenylpropanoid pathway in grapevine visualizing genes specifically modulated during ripening in four Corvina and four Garganega vineyards. The expression of these genes putatively leads to the biosynthesis of hydroxycinnamic and hydroxybenzoic acids, flavonols/dihydroflavonols, flavan-3-ols/proanthocyanidins, and resveratrol/stilbenes. Gene expression is represented as the log2 of the raw expression value normalized by row median for each cultivar separately. **(B)** Relative comparison of the levels of the phenolic compounds detected in the Garganega and Corvina berries by RP-HPLC-ESI-MS at veraison (stage 1), mid-maturity (stage2), and in ripe berries (stage 3).

The relative transcriptomic and metabolomic plasticity of Garganega and Corvina berries during ripening was determined by comparing gene expression and metabolite accumulation in the context of the phenylpropanoid/flavonoid pathway. The gene expression and phenolic profiles for each variety at veraison (stage 1), mid-maturity (stage 2), and in fully-ripe berries (stage 3; Supplementary File 4) are represented in Figures 7A,B, respectively.

Several differences between the varieties and locations were highlighted by this analysis. Some of the transcriptional trends changes were also confirmed by semi-quantitative Real Time RT-PCR analysis (Supplementary Figure 6). Garganega vineyard VH1 was characterized by a slight decline in the levels of hydroxycinnamic and hydroxybenzoic acids and the corresponding gene expression levels, whereas these compounds accumulated in the other three vineyards and the corresponding genes were induced. In particular, the caffeate 3-O-methyltransferase COMT2 (VIT_18s0072g00920) increased in vineyard AP, and the trans-cinnamate 4-monooxygenase VvC4H (VIT_06s0004g08150), which catalyzes the biosynthesis of p-coumarate from cinnamate, remained strongly expressed in vineyards VP and VH2 during ripening. The Garganega berries accumulated different amounts of these metabolites in different vineyards, mirroring the expression profiles of the corresponding genes. In contrast, a similar small decline in hydroxycinnamic and hydroxybenzoic acid levels was associated with Corvina berry ripening in all four vineyards, consistent with the overall downregulation of genes involved in the synthesis of these compounds. The hydroxycinnamic and hydroxybenzoic acid profiles differed substantially between the two varieties, and were more plastic in the white variety.

There was no substantial difference in resveratrol/stilbene accumulation in Garganega berries, even though stilbene synthases were upregulated in vineyard VH2, and the transcription factor VvMYB14 (VIT_07s0005g03340), which regulates the stilbene synthase gene family (Holl et al., 2013), was upregulated in vineyards VP and VH2. Stilbene accumulation correlated well with gene expression in Corvina berries, especially in the CC and PSP vineyards where the averaged expression level of stilbene synthase genes increased toward the final ripening stage.

The flavonol and dihydroflavonol content remained stable during the maturation of Garganega berries in vineyard VH1, whereas in the other vineyards a general increase in these compounds was observed. Flavonol synthase genes were upregulated during berry ripening in the VH2 vineyard and, to a lesser extent, in vineyards AP and VP. In contrast, flavonols and dihydroflavonols accumulated in a similar manner in the four Corvina vineyards. This may reflect the slight but consistent increase in the averaged expression level of flavonol synthase and flavonoid glucosyltransferase genes during ripening.

The flavan-3-ols and their oligomers declined marginally in both cultivars and in the different vineyards, especially VH1 which featured the strongest reduction among the Garganega vineyards. This correlated with the pronounced decline in the expression of *VvANR* (anthocyanidin reductase, VIT_00s0361g00040) in Corvina berries, and *VvLAR2* (leucoanthocyanidin reductase 2, VIT_17s0000g04150) in Garganega berries, but did not correlate with the expression of *VvLAR1* (leucoanthocyanidin reductase 1, VIT_01s0011g02960), which remained high at the third ripening stage in the Corvina vineyard PM and the Garganega vineyards VP and VH1. Interestingly, the expression profiles of the two MYB transcription factors regulating this branch of the flavonoid pathway differed in the two varieties, i.e., *VvMYBPA1* was expressed more strongly in Garganega berries, whereas *VvMYBPA2* was expressed more strongly in Corvina berries.

## Discussion

We used two large-scale analytical approaches to explore metabolomic and transcriptomic changes during the ripening of Garganega berries, a *V. vinifera* white berry variety. In order to understand the molecular basis of the environmental impact on berry ripening, four vineyards were selected to maximize differences in environmental conditions and to minimize differences due to agricultural practices such as the training system, orientation of the rows, planting layout, vineyard age, and rootstock.

The four selected growing sites belong to the same production area and were chosen to ensure diverse environmental parameters such as soil origin, texture, and composition. The soils in three of the vineyards were characterized by a low percentage of sand and a low concentration of calcium carbonate, as expected given their volcanic origin, and the fourth was alluvial in origin with a high proportion of clay. Vineyards at different altitudes were chosen to maximize the environmental variability. However, despite the distance of 400 m between the lowest (32 m above sea level) and highest (437 m above sea level) vineyards, the meteorological parameters recorded at the four sites revealed little difference in heat unit accumulation and rainfall during the 2013 growing season. Even so, the number of rainy days during the pre-blooming period (i.e., pre-flowering period) was greater at the VH2 site and there was a higher temperature during July, August, and September. This may have contributed to the specific ripening behavior we observed. Indeed, the pedoclimatic conditions and other uncontrolled variables strongly influenced the ripening dynamics at each site in terms of sugar accumulation and the synthesis of phenolic compounds. The most divergent ripening dynamics were characterized by an early decline in sugar accumulation (VH1) and a consistent high sugar accumulation rate (VH2).

The phenolic fraction of the Garganega berry metabolome was investigated by HPLC-MS. The phenolic composition of Garganega berries was found to be similar to that reported for another white berry cultivar, Albariño blanco (Di Lecce et al., 2014). One particularly relevant feature was the presence of a compound putatively identified as a myricetin derivative, a flavonol that is normally absent from *V. vinifera* white grapes (Flamini, 2013). The quantitation of berry metabolites during ripening showed that the levels of the various classes of phenolic compounds, especially hydroxycinnamic acids and flavonols, were highly variable at veraison in the different vineyards, and that they changed in different and unpredictable ways during the subsequent stages. In the plain vineyards, the relative levels of hydroxycinnamic acid increased rapidly from stage 1 (veraison) to stage 4 (maturity), whereas the relative levels of flavonols increased in three of the four vineyards. Overall these results suggest that the phenolic fraction in Garganega berries is highly responsive to the pedoclimatic conditions encompassed by our study.

The investigation of transcriptomic data by PCA revealed that the four growing sites strongly affected the dynamics of the ripening berry transcriptome. The distribution of samples based on the first two components showed that PC1 describes the changes associated with berry development, as seen in previous transcriptome surveys (Lijavetzky et al., 2012; Pastore et al., 2013). This evidence is supported by the presence of many “switch” genes, representing putative master regulators of the shift from immature to mature growth (Palumbo et al., 2014), among the positive loadings of PC1, i.e., those upregulated during ripening. Furthermore, the presence of several photosynthesis genes among the negative loadings of PC1, i.e., those downregulated during ripening, reflected the progressive shutdown of photosynthesis associated with berry ripening, and further supports the directional distribution of samples on PC1 during the transcriptome changes underlying general berry development. This interpretation suggests that, despite the minor differences in PC1-values among samples collected at veraison, the ripening process was distinct in the four vineyards, with the greatest differences observed between high-altitude sites. Indeed, VH1 berries accumulated the lowest levels of TSS at harvest and yielded the lowest PC1-values, whereas VH2 berries, the richest in sugars, achieved the highest PC1-values at harvest. The distribution of samples along PC2 highlighted different and sometimes divergent behaviors accounting for the highly plastic responses of Garganega berries to the pedoclimatic conditions at the four growing sites. Interestingly, by focusing on the first and last percentiles of the PC2 loadings, we found that such plastic responses included the differential expression of genes mainly belonging to the same functional categories already assigned to plastic genes modulated in Corvina berries, e.g., DNA/RNA metabolic processes, Transport, Carbohydrate metabolism, Cellular processes, and Homeostasis (Dal Santo et al., 2013a). The large number of genes in the DNA/RNA metabolic process and transcriptional regulation categories in both cultivars strongly supports a key role for transcriptional and translational control in the transcriptomic plasticity of ripening berries (Dal Santo et al., 2013a). PC2 also contained several genes involved in carbohydrate metabolism, in particular genes encoding enzymes required for anaerobic metabolism (e.g., pyruvate decarboxylase, alcohol dehydrogenase, and aldehyde dehydrogenase), and those related to glycolysis and malic acid metabolism (e.g., pyruvate kinase, phosphoenolpyruvate carboxylase, malate dehydrogenase, and malic enzyme).

Although we did not observe a clear relationship between the genes and enzymes responsible for malate metabolism, the direct influence of environmental conditions on malate metabolism was extensively characterized in early studies (Lakso and Kliewer, 1975, 1978) and also more recent studies (Sweetman et al., 2009, 2014) of berries ripening under diverse temperature regimes. The differential expression of genes involved in malate metabolism in Garganega berries grown at different sites may therefore reflect the distinct light and temperature conditions at each location.

Our investigation allowed to explore plasticity by identifying genes specifically modulated in each of the four vineyards. This revealed that VH2, characterized by accelerated ripening, featured the largest number of specifically modulated genes, especially several abiotic and biotic stress response genes whose expression was often associated with the berry maturation process (Tornielli et al., 2012) Secondary metabolism responded strongly to the environment particularly at the two high-altitude vineyards. Genes representing terpenoid, lignin, and carotenoid metabolism, as well as genes encoding GSTs, were all specifically modulated in vineyard VH2, whereas stilbene metabolism was most strongly affected in vineyard VH1.

The plasticity of phenylpropanoid metabolism is a well-known feature of grape berries and this confers many of the wine quality traits that represent specific terroirs (Teixeira et al., 2013). A strong correlation between phenylpropanoid accumulation and the expression of corresponding genes has recently been reported in Corvina berries (Dal Santo et al., 2013a; Anesi et al., 2015). The analysis of Sauvignon Blanc berries has likewise shown that carotenoid metabolism is highly responsive to the microclimate (Young et al., 2015). The analysis of carotenoid-related genes in Garganega berries revealed highly divergent expression profiles in the four vineyards, supporting the plastic behavior of this class of compounds which function to protect the photosynthetic membranes, promote the synthesis of ABA and strigolactone, and to generate volatile flavor/aroma compounds (Young et al., 2012).

The relative plasticity of Garganega berries at different sites was compared to the previously reported transcriptomic and metabolomic plasticity of Corvina berries (Dal Santo et al., 2013a; Anesi et al., 2015). We compared the number of differentially expressed genes detected when Corvina berries were ripened under four different environmental conditions and identified the environmentally sensitive portion of the developmentally regulated transcriptome. This revealed that the proportion of specifically modulated genes is similar in Corvina and Garganega berries, and many plastic genes are shared between the two varieties. We then focused on genes and metabolites involved in the phenylpropanoid/flavonoid pathway. The Garganega berries revealed vineyard-related trends in the accumulation or depletion of metabolites, whereas Corvina samples from all four sites showed highly similar metabolic trends during ripening, with a slight decline in the level of hydroxycinnamic acids and an increase in the levels of anthocyanins, flavonols, and stilbenes. The unique behavior of the Garganega cultivar, which increased (AP, VP), reduced (VH1), or maintained (VH2) the total level of phenolic compounds during ripening, together with the greater metabolomic diversity at veraison compared to Corvina berries, strongly suggests that Garganega is much more plastic than Corvina in terms of the accumulation of phenolic compounds during ripening in different environments. Metabolic differences between the two varieties were also strongly supported by the consistency of the expression profiles of phenylpropanoid/flavonoid related genes during berry maturation.

In conclusion, this study provides an overview of the transcriptomic and metabolomic responses to different growing sites in Garganega, a white grapevine variety cultivated in the eastern hills of the Verona province. The typicity of Garganega wines depends on the unique effect of the growing area and climatic conditions on the grapevine genotype. This variety is therefore an excellent model to dissect the molecular mechanisms underlying *terroir*-dependent quality traits in wines and to improve the interpretation of phenotypic plasticity in grapevine. The sensitivity of phenylpropanoid/flavonoid metabolism to the Garganega growing site supports previous data based on the analysis of red cultivars highlighting the important role of phenolic plasticity in the investigation of plasticity in general. In this context, our results could help to define how different varieties interact with the environment to promote the accumulation of phenolic compounds, and thus help with the development of strategies to cope environmental changes or to enhance the phenolic composition of wines.

## Author contributions

SDS and MF interpreted the bioinformatics data, coordinated the study, and wrote the manuscript. SN carried out the metabolomics analysis and helped to draft the manuscript. ED performed the microarray experiments and the qPCR analysis. MP contributed with supervision and coordination expertise. FG helped to interpret the metabolomics data and draft the manuscript. NV contributed to the design of the study. GBT participated in the design of the study, interpreted the microarray data and drafted the manuscript. SZ conceived, designed and supervised the study and wrote the manuscript.

## Funding

This work was supported by Joint Project 2014, funded by the Regione Veneto, “Innovative molecular approaches to investigate the interaction between cultivar Garganega and Soave volcanic soil” between Consorzio Tutela Vini Soave e Recioto di Soave (Soave, Verona, Italy) and the Biotechnology Department of University of Verona. The INNOVINE European Project FP7-311775 “Combining innovation in vineyard management and genetic diversity for a sustainable European viticulture” also supported the present work which benefited from the networking activities coordinated under the EU-funded COST ACTION FA1106 “An integrated systems approach to determine the developmental mechanisms controlling fleshy fruit quality in tomato and grapevine.” SDS was financially supported by the Italian Ministry of University and Research FIRB RBFR13GHC5 project “The epigenomic plasticity of grapevine in genotype per environment interactions.”

### Conflict of interest statement

The authors declare that the research was conducted in the absence of any commercial or financial relationships that could be construed as a potential conflict of interest. The reviewer CR and handling Editor declared their shared affiliation, and the handling editor states that the process nevertheless met the standards of a fair and objective review.
