# Supplementary figures and images for: Plasticity of the Berry Ripening Program in a White Grape Variety
Source: Front Plant Sci. 2016 Jul 12;7:970. doi: 10.3389/fpls.2016.00970 (PMC4940403; doi:10.3389/fpls.2016.00970)

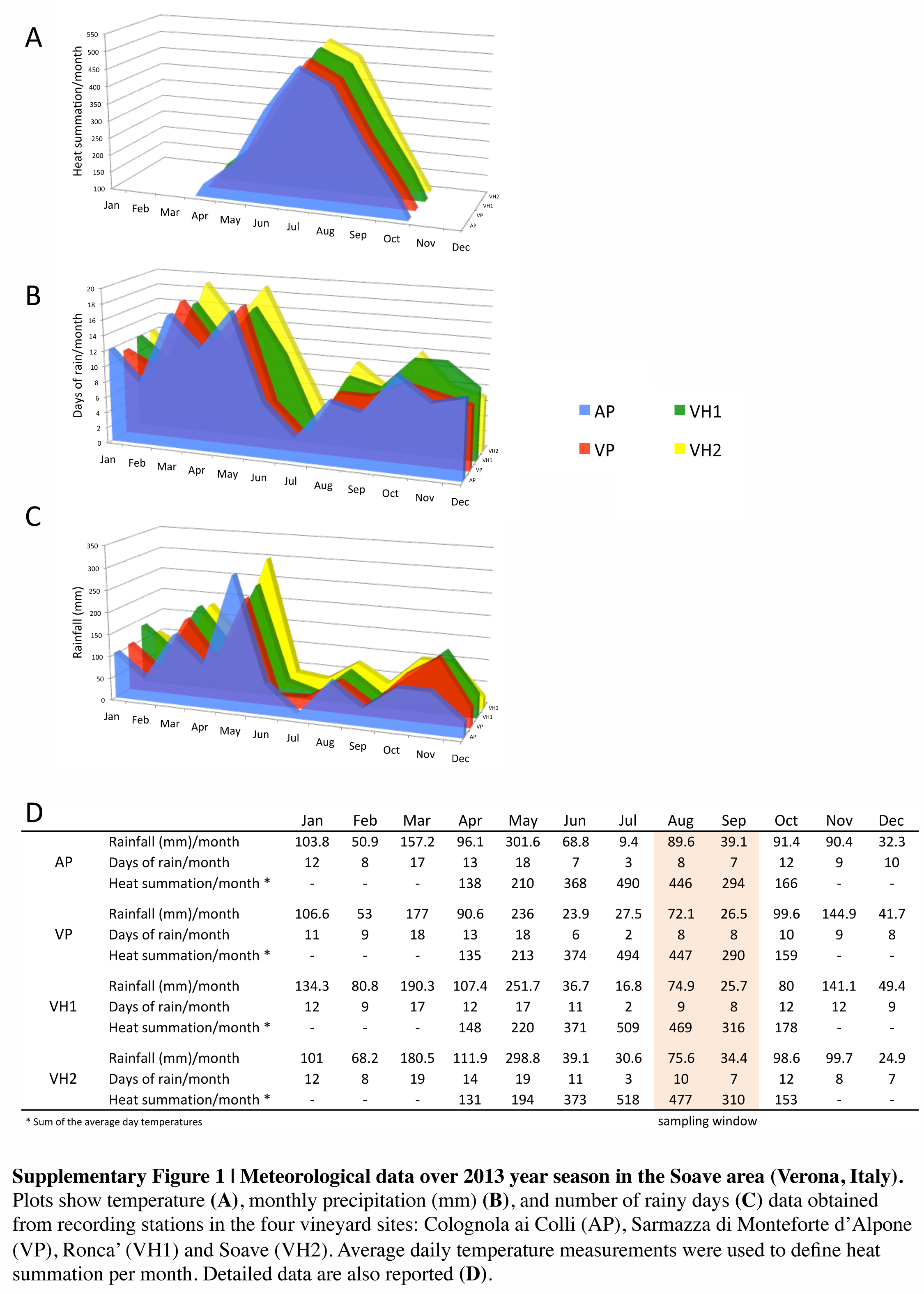

Supplement: Supplementary file 7 [file Image1.TIF]

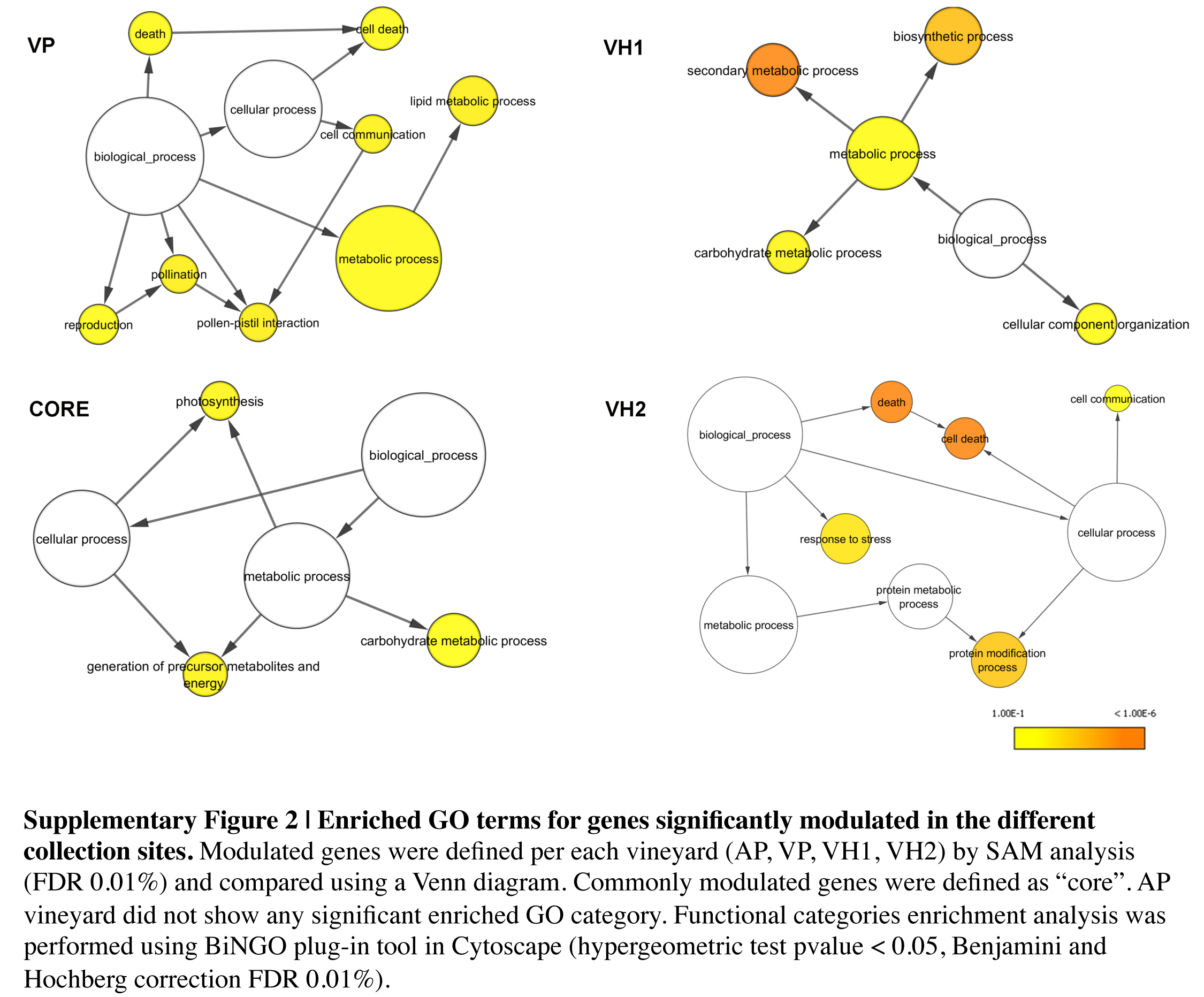

Supplement: Supplementary file 8 [file Image2.TIF]

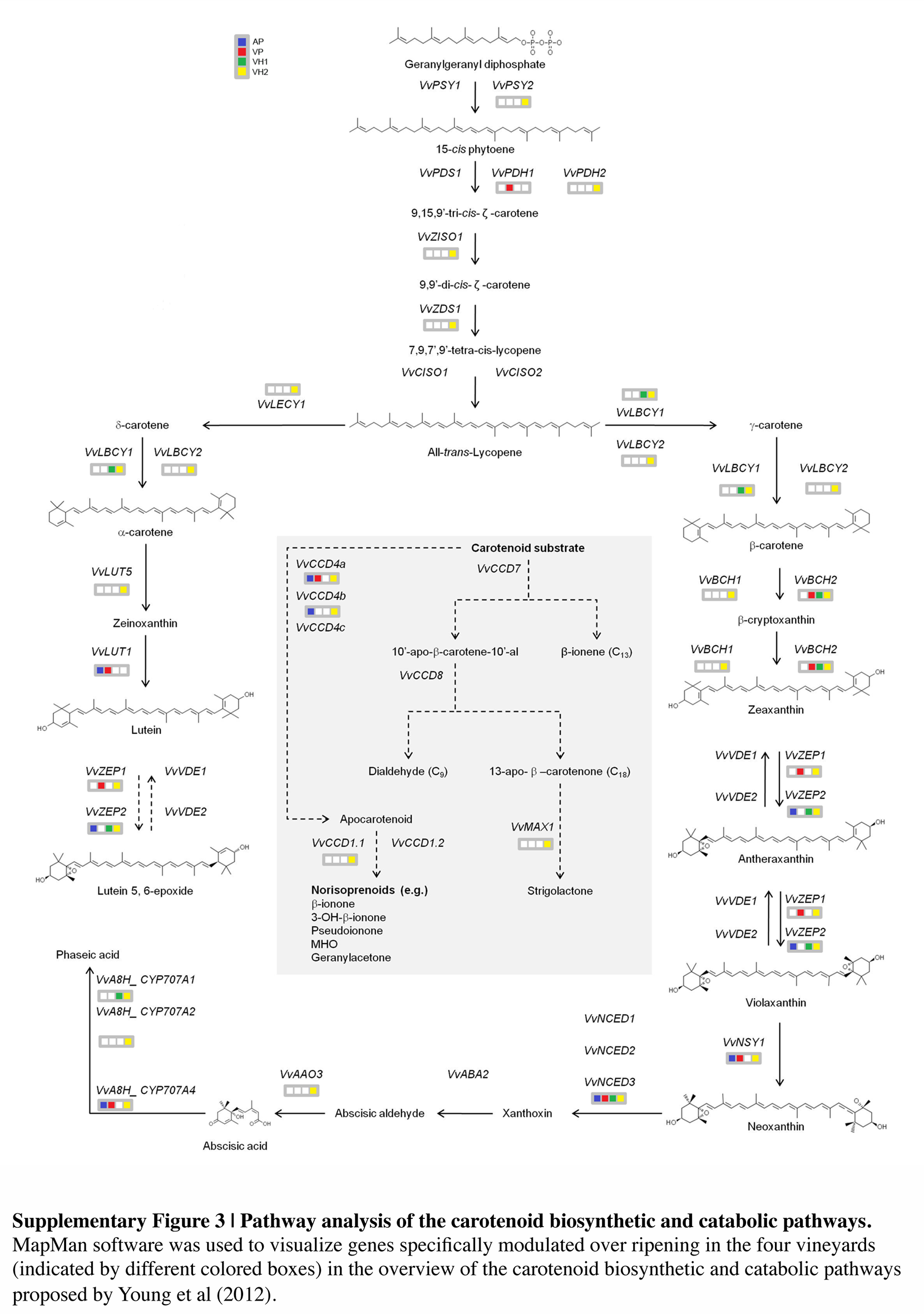

Supplement: Supplementary file 9 [file Image3.TIF]

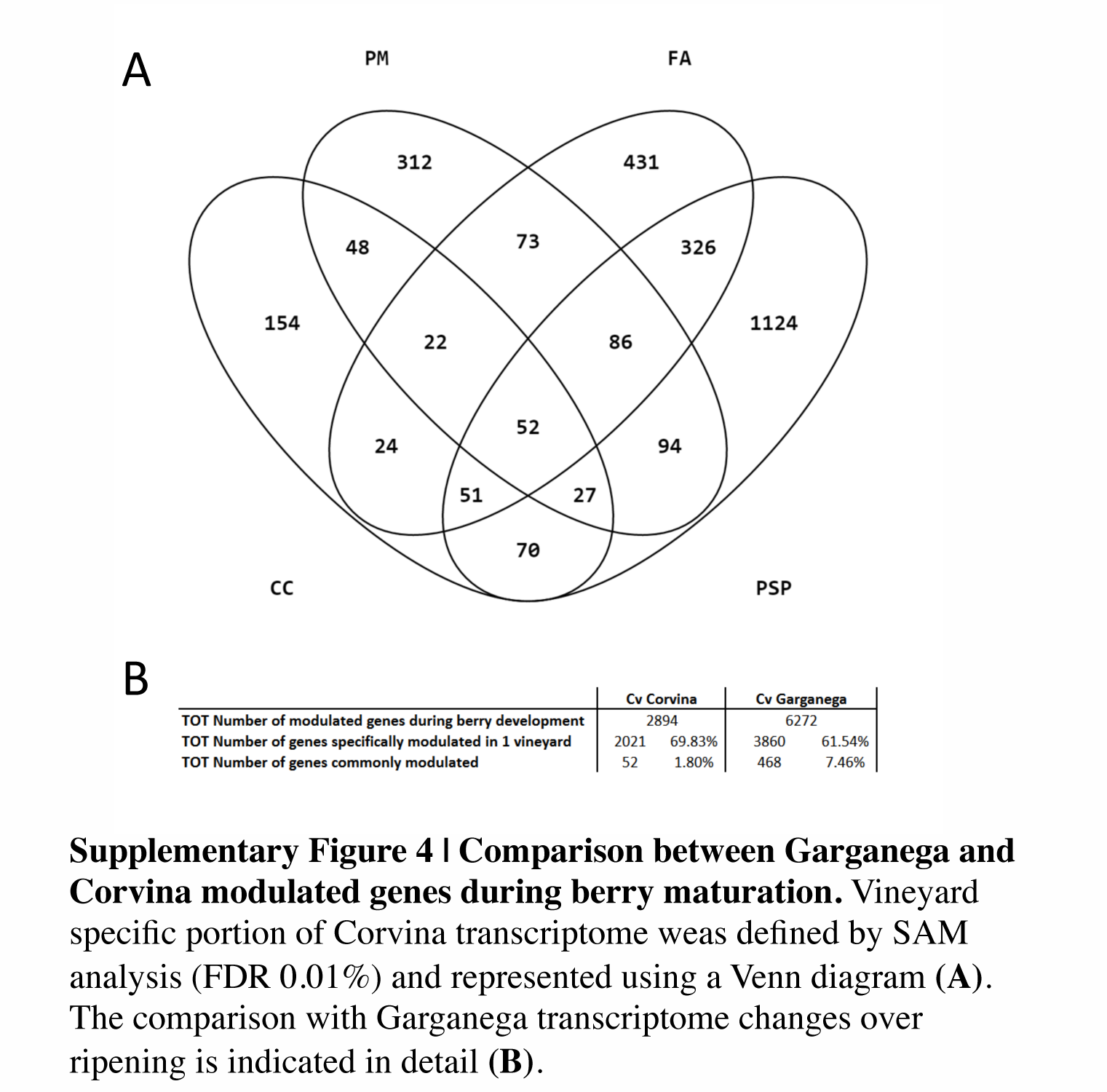

Supplement: Supplementary file 10 [file Image4.TIF]

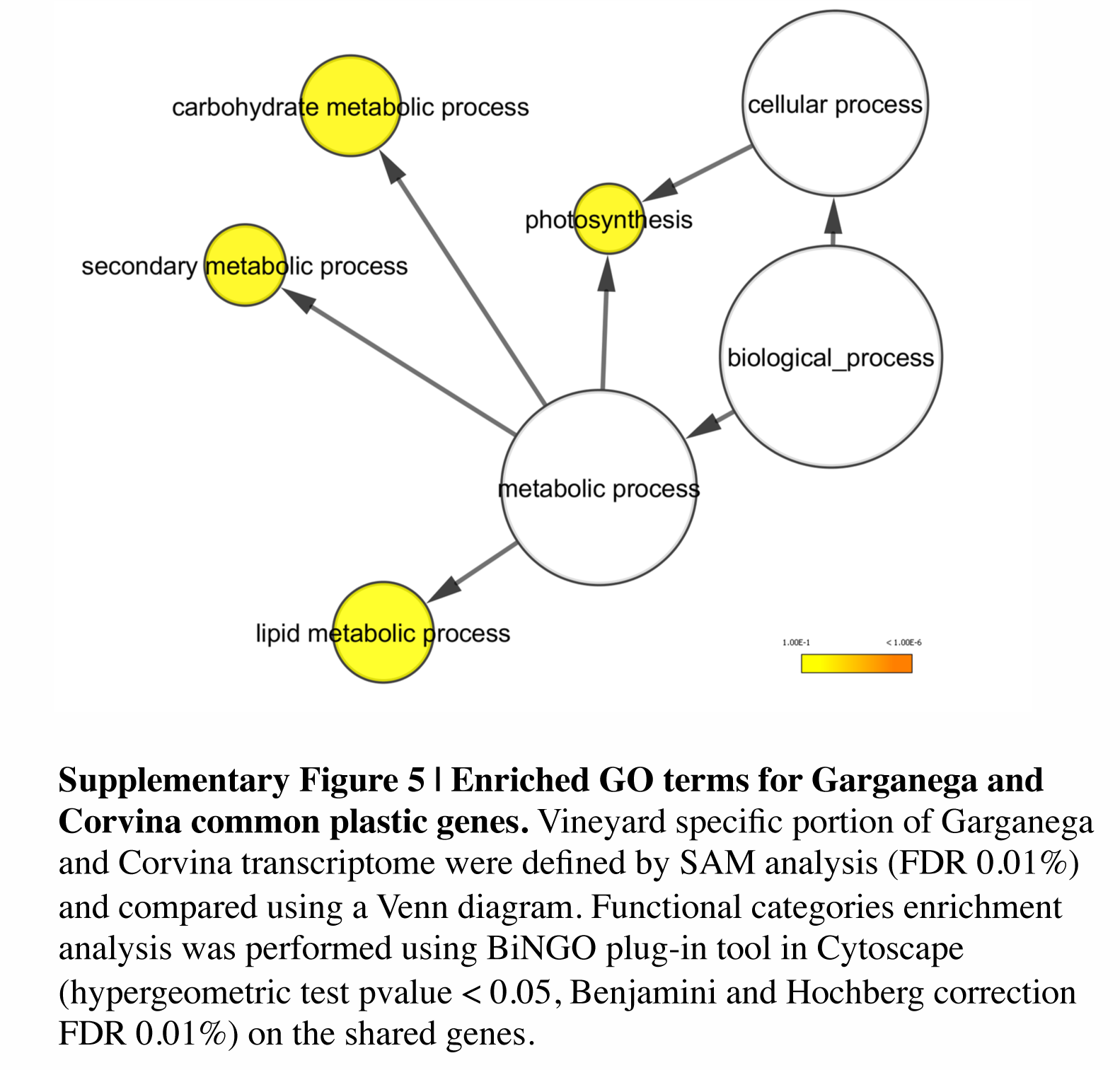

Supplement: Supplementary file 11 [file Image5.TIF]

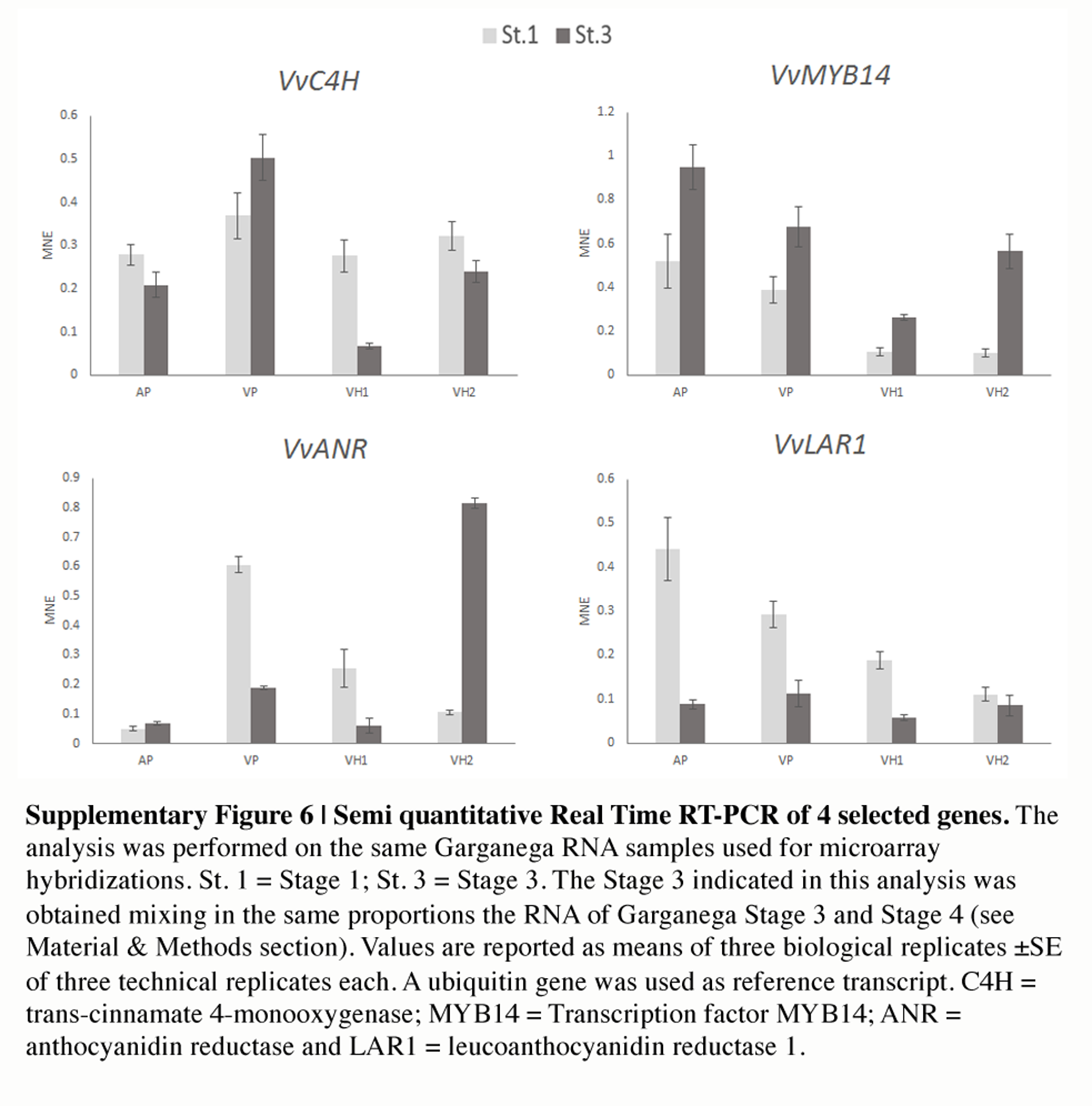

Supplement: Supplementary file 12 [file Image6.TIF]
